# Commentary: Functional Neuronal CB2 Cannabinoid Receptors in the CNS

**DOI:** 10.2174/157015911795017416

**Published:** 2011-03

**Authors:** E.S Onaivi

**Affiliations:** William Paterson University, Wayne NJ and Molecular Neurobiology Branch, National Institute on Drug Abuse, NIH, Baltimore, USA

**Keywords:** Cannabinoid, CB1, CB2, endocannabinoids, CNS, immune system, cannabis, marijuana.

## Abstract

Cannabinoids are the constituents of the marijuana plant (Cannabis sativa). There are numerous cannabinoids and other natural compounds that have been reported in the cannabis plant. The recent progress in marijuana-cannabinoid research include the discovery of an endocannabinoid system with specific genes coding for cannabinoid receptors (CBRs) that are activated by smoking marijuana, and that the human body and brain makes its own marijuana-like substances called endocannabinoids that also activate CBRs. This new knowledge and progress about cannabinoids and endocannabinoids indicate that a balanced level of endocannabinoids is important for pregnancy and that the breast milk in animals and humans has endocannabinoids for the growth and development of the new born. There are two well characterized cannabinoid receptors termed CB1-Rs and CB2-Rs and these CBRs are perhaps the most abundant G-protein coupled receptors that are expressed at high levels in many regions of the mammalian brain. The expression of CB1-Rs in the brain and periphery and the identification of CB2-Rs in immune cells and during inflammation has been extensively studied and characterized. However, the expression of functional neuronal CB2-Rs in the CNS has been much less well established and characterized in comparison to the expression of abundant brain CB1-Rs and functional neuronal CB2-Rs has ignited debate and controversy. While the issue of the specificity of CB2-R antibodies remains, many recent studies have reported the discovery and functional characterization of functional neuronal CB2-Rs in the CNS beyond neuro-immuno cannabinoid activity.

## FUNCTIONAL NEURONAL CB2 CANNABINOID RECEPTORS IN THE CNS

Cannabinoids are the constituents in marijuana and endocannabinoids (eCBs) are the endogenous marijuana-like substances found in animals and humans [1]. Recent advances in cannabinoid research indicate the existence of an endocannabinoid system (ECS) consisting of genes encoding cannabinoid receptors (CB1-Rs and CB2-Rs), their endogenous ligands eCBs and proteins that synthesize and degrade these eCBs. Both CB1-Rs and CB2-Rs are distributed in the brain and peripheral tissues and are activated by endocannabinoids, and cannabinoids, the active constituents in marijuana [1]. For many years it was thought that marijuana use, phytocannabinoids and eCBs act by activating brain-type cannabinoid receptors (CBRs) called CB1-Rs, and that a second type called CB2-Rs were found in peripheral tissues and mainly in immune cells and were referred to as peripheral CB2-Rs. This was because many investigators were not able to detect the presence of neuronal CB2-Rs in healthy brains [2-4], but CB2-R expression were demonstrated in rat microglia cells and other brain-associated immune cells during inflammation [5-10]. Despite the evidence that CB2-Rs might be present in the CNS, the expression of neuronal CB2-Rs in the CNS has been much less well established and characterized in comparison to the expression of abundant brain CB1-Rs. We and others and many recent studies have reported the discovery and functional characterization of neuronal brain CB2-Rs. A number of studies from mice to human subjects, using a variety of techniques including those used in pain models, histological, immunohistochemical, *in situ* hybridization, electron microscopy, molecular biological, behavioral and pharmacological, pharmacological MRI, cerebral occlusion and hemicerebellectomy, transgenic and cell culture studies show the functional presence of CB2-Rs in neural progenitor cells, neurons, glial and endothelial cells [11-14].

These functional neuronal CB2-Rs have ignited debate and controversy, and its possible involvement in drug addiction and neuropsychiatric disorders is intensely under re-evaluation. While the role of CB2-Rs in CNS disturbances involving neuroinflammation and neuropathic pain have been extensively reported [15], our studies provided the first evidence for a role of CB2-Rs in depression, schizophrenia and in the effects of substance abuse [15, 16 and 17]. The controversy of the functional expression of brain neuronal CB2-Rs remain because *CNR2* gene and CB2-Rs have received much less attention than CB1-Rs. Although the expression of CB1-Rs in the brain and periphery has been well studied, many features of *CNR2* gene structure, regulation and variation remain poorly characterized in comparison to the *CNR1* gene encoding the CB1-Rs. This poor characterization of *CNR2* gene structure and variants hampers progress in the determination of the functional role of CB2-Rs in a number of CNS disturbances. Additionally, the CNS presence of CB2-Rs may no longer be a debate, but the neurobiological basis for CB2-R physiological activity and its interaction with or without CB1-Rs remains to be determined. Therefore, an overwhelming number of studies now document CB2-R expression in neuronal, endothelial and glial cells. Mounting evidence also shows that CB2-Rs and its gene variants may play possible roles in neuroinflammation occurring in multiple sclerosis, traumatic brain injury, HIV-induced encephalitis, Alzheimer’s, Parkinson’s and Huntington’s diseases [18 and 19]. A central neuronal but glial independent neuroprotection by CB2-R activation was reported to counteract apoptotic cell death that is induced by remote axonal damage that is achieved through PI3K/Akt signaling [14]. Functional interactions between forebrain CB2-R and mu-opioid receptor (MOR) was demonstrated [20] and CB2-R antagonist SR144528 was reported to decrease MOR expression and activation in mouse brainstem [21]. Upon our discovery of the presence and functional expression of cannabinoid CB2-Rs in the brain [22], most recent studies have confirmed that CB2-Rs are present in both cultured neural cells and the nervous system of several mammals such as rodents, monkeys and humans under normal conditions [23]. Thus CB2-Rs have been implicated in the control of fundamental neural cell processes, such as proliferation and survival. It was therefore suggested that manipulating CB2-Rs might be useful for delaying the progression of neurodegenerative disorders and inhibiting the growth of glial tumors [23]. CB2-Rs have also been shown sub-serving differential physiological roles in other neuroanatomical sites such as the brain stem, cortex, cerebellum, PAG, substantia nigra, hippocampus, thalamus, pineal gland and pinealocytes [7, 8, 24-27]. CB2-Rs in the pineal gland along with other components of the ECS may be involved in the control of pineal physiology [28]. Gender-dependent changes on the expression of hippocampal CB1 and CB2-Rs were demonstrated in the early maternal deprivation model in neonatal rats [27]. While CB1-Rs remain one of the most ubiquitous G-protein coupled receptors in the mammalian brain, we have described the multifocal distribution of CB2-Rs albeit at lower levels than the CB1-Rs in neuronal and glial processes in a number of brain areas [25]. This multifocal distribution and the presence of CNS brain CB2-Rs suggest a need to re-evaluate the role of these receptors in neurotransmission. It is important to understand the role of CB2-Rs and its gene variants in the CNS and its possible involvement drug addiction and neuropsychiatric disorders. Research however on the involvement of CB2-Rs in neuroinflammatory conditions and in neuropathic pain has advanced more than other areas in neuropsychiatry and drug addiction. Therefore, improved information about *CNR2* gene and its human variants might add to our understanding, not only of the role of CB2-Rs during neuroinflammatory conditions in the CNS but also beyond neuro-immuno-cannabinoid activity.

Many previous studies could not detect the expression of CB2-Rs in the brain [2, 4 and 29 ], because the PCR primers may not have been specific to detect CB2-R isoforms. Furthermore, the specificity of the available antibodies for both CB1 and CB2-Rs has also been controversial as some could not detect the native and in some cases the transfected cannbinoid CBR antigen, although they recognized proteins in Western blot and in immunohistochemical analysis, [33]. There are also problems with the antibodies because of the species differences between human and rodent CB2 gene. We have resolved some of these issues by using CB2 isoform specific TaqMan probes that could differentiate the isoform-specific expression and are more sensitive and specific than CB2 antibodies that are currently available [32]. The controversial CB2-R brain expression could also be due to the low expression levels of CB2A isoform in brain regions and the less specific CB2 commercial antibodies in immunohistochemical studies, especially those studies using antibodies against human hCB2 epitopes for rodent brain immunostaining. There are also problems with the use of the CB2 knockout (ko) [34] mice in Western blots and in behavioral analysis. When we analyzed the CB2 knockout mice using the three TaqMan probes against two promoters of mouse CB2 gene and the deleted part of CB2 gene, we found that the promoters of CB2-R ko mice were still active and that a CB2 truncated version was expressed, indicating that the CB2 ko mice with ablation of the C-terminal peptides of 131 amino acids [34] was an incomplete CB2-R knockout [32]. Another mouse CB2-R ko mice that has now been generated with ablation of N-terminal peptide 156 amino acid (Deltagen, Inc. San Mateo, CA) may clarify the specificity of the antibodies that were used against the N-terminal epitopes. Thus, contrary to prior reports that CB2-Rs were not expressed in the brain, we and others have now reported the wide distribution of CB2-Rs in brain regions, suggesting a re-evaluation of the role of CB2-Rs in the CNS.

The complete gene structure, 5’- and 3’ –UTR, and transcription initiation sites of human CB2-Rs have not been fully characterized [22, 31], until now. After we and others identified and reported mouse CB2-R expression in brain regions [24 and 25], the specific expression of human or mouse CB2-R isoforms in brain regions was not known. But the published evidence showed significant species differences of CB2-Rs in human, mouse and rat in terms of peptides, mRNA sizes, gene structure and pharmacology [2, 29 and  30]. Therefore, the discrepancies on the CB2-R mRNA sizes in the literature indicated incomplete gene structure of *CB2* gene in different species or polymorphism in the same species. We have discovered a novel human *CNR2* gene promoter encoding testis (CB2A) isoform starting exon located ca 45 kb upstream from previously identified promoter encoding the spleen isoform (CB2B) [32]. The size of the newly identified hCB2A isoform is about twice as large as the previously identified human hCB2B gene. The 5’ exons of both CB2-R isoforms are untranslated 5’UTRs and alternatively spliced to the major protein coding exon of *CNR2* gene. We found that CB2A is expressed higher in testis and brain than CB2B that is expressed higher in other peripheral tissues than CB2A. Using precise probes and species comparison, we found that the *CNR2* gene of human, rat and mouse genomes deviated in their gene structures and isoform expression patterns and could be regulated by cannabinoid ligand treatment in the mouse model [32]. The human CB2 gene is almost four times larger than the mouse and rat CB2 genes [32]. If the transcription rates are similar between human and rodents, hCB2A isoform would take much longer time to be transcribed in the testis and brain. This will be unusual because other gene orthologs between humans and mice are usually within one fold difference in genomic sizes. Our data shows that there are two forms of the CB2-Rs in human, rat and mouse with differential subtype distribution specificities in the brain and peripheral organ tissues [32]. The promoter-specific CB2-R isoform distribution may in part explain why CB2-Rs were previously undetectable in both human and rodent brains [2, 4 and 29]. Several other functional studies reveal roles for CB1-Rs and CB2-Rs [34-44]. However, our studies provided the first evidence for the CNS effects of CB2-Rs and its possible involvement in drug addiction and neuropsychiatric disorders [15-17, 45 and 46]. We utilized behavioral and molecular methods to study and determine whether there was a link between depression that may be a factor in drug/alcohol addiction and CNS CB2-Rs. First we established the use of mouse chronic mild stress (CMS) model of depression which has been validated and a widely used model for screening anti-depressants. Briefly the mouse CMS model measures one of the core symptoms of depression which is anhedonia, a lack of pleasure. Then, mice were subjected daily for four weeks to CMS, and anhedonia was measured by the consumption of sucrose solution. Behavioral and rewarding effects of abused substances were determined in the CMS and control animals. The expression of CB2-Rs and their gene transcripts was compared in the brains of CMS and control animals by Western blotting and RT-PCR. CMS induced gender-specific aversions in the test of anxiety which were blocked by WIN55212-2 and a CB2-R agonist. In other studies we demonstrated that direct CB2-R antisense oligonucleotide microinjection into the mouse brain induced anxiolysis, indicating that CB2-Rs are functionally present in the brain and may influence behavior [15-17, 45 and 46]. In another recent study, using *in situ* hybridization and PCR and newly designed CB2-R mRNA probes [32], to avoid the controversial CB2-R antibodies, the existence of CB2-R transcripts was demonstrated in a variety of brain areas of the primate *Macaca fascicularis*, including the cerebral cortex, hippocampus and in both external and internal divisions of the globus pallidus [47]. 

The clinical and functional implication of neuronal CB2-Rs in the brain will gradually become clearer because more research will certainly unravel the contribution and interaction of CB1 and CB2-Rs in drug addiction, neuropsychiatry and neurodegenerative disorders. The new knowledge from our data and those of other recent studies that CB2-Rs are functionally present in neurons, in the brain raises many questions about the possible roles that CB2-Rs may play in the nervous system. These results therefore extend the previous evidence that CB2-Rs are playing an important role in immune function to other putative neuronal function by their apparent presence in neuronal processes. Our studies implicate neuronal and glial CB2-Rs in the chronic mild stress model of depression, and substance abuse. The immunohistochemical localization of CB2-Rs, when compared to that of known CB1-R distribution in the brain, may be an indication of other putative functional roles of CB2-Rs in the CNS. Therefore both CB1 and CB2 receptors seem likely to work both independently and/or cooperatively in differing neuronal populations to regulate important physiological activities in the central nervous system. Recent events in the clinic have linked the use of the anti-obesity drug, acomplia, a CB1-R antagonist and an appetite suppressant with a higher risk of depression and suicide. Our data have also showed associations of the *CNR2 *gene with depression, drug abuse, anorexia nervosa and schizophrenia in a human population and also in the CMS model of depression [17,  45] suggesting that the CB2-Rs may be involved in the endocannabinoid signaling mechanisms associated with the regulation of emotionality beyond immune function. More studies are therefore required to determine if CB2-R ligands have the risk of depression or suicide that has led to the withdrawal of acomplia, (rimonabant) from use as an appetite suppressant in the control of obesity in Europe.
